# Human adenovirus among hospitalized children with respiratory tract infections in Beijing, China, 2017–2018

**DOI:** 10.1186/s12985-019-1185-x

**Published:** 2019-06-13

**Authors:** Li-hong Yao, Chao Wang, Tian-li Wei, Hao Wang, Fen-lian Ma, Li-shu Zheng

**Affiliations:** 1 NHC Key Laboratory of Medical Virology and Viral Diseases, National Institute for Viral Disease Control and Prevention, China CDC, 100 Ying-Xin St., Xi-Cheng District, Beijing, 100052 China; 2 Department of Pediatrics, Beijing Friendship Hospital, Capital Medical University, Beijing, 100050 China

**Keywords:** Human adenovirus, Respiratory tract infection, Epidemiology

## Abstract

**Background:**

Human adenoviruses (HAdVs) cause a wide range of diseases. However, the genotype diversity and epidemiological information relating to HAdVs among hospitalized children with respiratory tract infections (RTIs) is limited. Here, we describe the epidemiology and genotype distribution of HAdVs associated with RTIs in Beijing, China.

**Methods:**

Nasopharyngeal aspirates (NPA) were collected from hospitalized children with RTIs from April 2017 to March 2018. HAdVs were detected by a TaqMan-based quantitative real-time polymerase chain reaction (qPCR) assay, and the hexon gene was used for phylogenetic analysis. Epidemiological data were analyzed using statistical product and service solutions (SPSS) 21.0 software.

**Results:**

HAdV was detected in 72 (5.64%) of the 1276 NPA specimens, with most (86.11%, 62/72) HAdV-positives cases detected among children < 6 years of age. HAdV-B3 (56.06%, 37/66) and HAdV-C2 (19.70%, 13/66) were the most frequent. Of the 72 HAdV-infected cases, 27 (37.50%) were co-infected with other respiratory viruses, most commonly parainfluenza virus (12.50%, 9/72) and rhinovirus (9.72%, 7/72). The log number of viral load ranged from 3.30 to 9.14 copies per mL of NPA, with no significant difference between the HAdV mono- and co-infection groups. The main clinical symptoms in the HAdV-infected patients were fever and cough, and 62 (86.11%, 62/72) were diagnosed with pneumonia. Additionally, HAdVs were detected throughout the year with a higher prevalence in summer.

**Conclusions:**

HAdV prevalence is related to age and season. HAdV-B and HAdV-C circulated simultaneously among the hospitalized children with RTIs in Beijing, and HAdV-B type 3 and HAdV-C type 2 were the most frequent.

## Background

First discovered in 1953 by Rowe et al., human adenoviruses (HAdVs) are non-enveloped, double-stranded DNA viruses belonging to the Mastadenovirus genus (Adenoviridae family). They are common pathogens in children and cause a variety of diseases [1]. HAdV accounts for at least 5 to 10% of pediatric and 1 to 7% of adult respiratory tract infections (RTIs) [2]. There are currently seven different HAdV species (A to G), some of which specifically attack the conjunctiva (species D), the upper and lower respiratory tracts (species B, C and E), and the gastrointestinal tract (species F and G) [3]. Of the 90 AdV serotypes, 55 are known to cause human diseases, with AdV3, 4, 7 and 14 being the most common types to cause respiratory disease outbreaks [4]. HAdV has caused respiratory tract adenovirus outbreaks in Jiangsu and Taiwan provinces of China, as well as in Korea, Singapore and Malaysia [5–7]. Such infections have been estimated to cause 2–5% of RTIs overall and 4–10% of all pneumonias in City of Bethlehem [8].

Although HAdVs are associated with mild to moderate disease in most cases, life threatening disease can occur in some patients, particularly if they are immunocompromised [9]. At present, China has not yet established a nationwide epidemiological surveillance program for adenovirus infections, and infections with it do not need to be legally reported, so the institutions for disease control and prevention cannot conduct early detection screening or issue early warnings. Neither are there any U.S. Food and Drug Administration approved antivirals for adenoviral infections [10]. HAdVs play an important role in respiratory infections, particularly in children. Therefore, the aim of this study was to evaluate the epidemiological, clinical, and molecular characteristics of HAdV infections occurring among children with RTIs in a Chinese tertiary hospital from April 2017 to March 2018. Collectively, the findings from this study underscore the importance of monitoring the epidemiology of HAdV infections and protecting vulnerable patients as part of the suite of infection prevention strategies in hospitals.

## Materials and methods

### Patients and specimens

The nasopharyngeal aspirate (NPA) samples (1276) used in this study were collected from hospitalized children (< 14 years) with RTIs at Beijing Friendship Hospital between April 2017 and March 2018. Informed consent from the parents or guardians of the children enrolled in the study was received. RTIs was defined as an illness that presented with at least two of the following clinical presentations: fever, cough, nasal obstruction, expectoration, sneeze and dyspnoea during the previous week. Patients, who were diagnosed with pneumonia by chest radiography, were also included in the study, even if they did not show the clinical features described above [11]. All the specimens were stored at − 80 °C until further processing. Demographic and clinical data were obtained from the hospital’s database.

### Detection of HAdV and other common respiratory viruses

Total viral nucleic acids were extracted from 200 μL of each clinical NPA specimen using the QIAamp MinElute Kit (Qiagen, Germany) according to the manufacturer’s instructions and eluted with 60 μL of nuclease-free water. HAdV detection was performed using qPCR assay targeting the highly conserved gene region (132-bp) of the HAdV hexon. TaqMan Universal PCR Master Mix (Applied Biosystems, USA) was used to amplify HAdV hexon DNA using specific primers (Forward:5′- GCCCCAGTGGTCTTACATGCACATC-3′; Reverse: 5′-GCCACGGTGGGGTTTCT AAACTT-3′) and probe (5′-FAM-TGCACCAGACCCGGGCTCAGGTACTCCGA- 3′-TAMRA) [12]. Each 20 μL reaction mixture comprised 10 μL of 2 × TaqMan Gene Expression Master Mix, 1.8 μL of each primer (10 pM), 0.2 μL of probe (10 pM), 4.2 μL of sterile water, and 2.0 μL of the nucleic acid components extracted from each sample. The qPCR cycling program was as follows: 50 °C for 2 min, 95 °C for 10 min, followed by 40 cycles of 95 °C for 15 s, and 60 °C for 1 min. Samples with a cycle threshold (Ct) < 38 were regarded as positive. 10-fold serial dilutions of pMD18-T/Hexon plasmid from 10^10^ to 10^0^ copies per μL were prepared to establish a standard curve to measure the HAdV load. qPCR were performed using the Mx3005P qPCR System (Agilent Stratagene, USA). HAdV-positive samples were subsequently screened for the following pathogens: influenza A (FluA) and B (FluB) viruses, parainfluenza viruses (PIVs) 1–4, human metapneumovirus (HMPV), human rhinovirus (HRV), WU polyomavirus (WUPyV), respiratory syncytial virus (RSV) and human coronaviruses (HCoV) NL63, OC43, 229E, HKU1 and human bocavirus (HBoV), as described previously [13–15]. Additionally, mycoplasma pneumonia is determined by the detection of mycoplasma pneumoniae IgM antibody. As for bacterial pneumonia, it was confirmed by sputum culture.

### HAdV genotyping

Nested PCR targeting the HAdV hexon gene’s hypervariable region was employed for genotyping. The outer primers used were forward 5′-GCCACCTTCTTCCCCATGGC.

− 3′ and reverse 5′-GTAGCGTTGCCGGCCGAGAA-3′, and the internal primers were forward 5′-TTCCCCATGGCCCACAACAC-3′ and reverse 5′-GCCTCGATGACGCC.

GCGGTG-3′. Nested PCR was conducted in 25 μL volume comprising 2.5 μL of 10 × ^EX^Taq buffer, 1.0 μL (10 pM) of each primer, 2.0 μL of dNTP Mix, 0.5 μL of ^EX^Taq DNA polymerase, 2.0 μL of viral nucleic acid extract or first nested-PCR product, and 16 μL of double-distilled water. The mixtures were amplified with an initial denaturation at 94 °C for 10 min, followed by 36 cycles at 94 °C for 1 min, 55 °C for 1 min, and 72 °C for 2 min, and a 7 min extension at 72 °C. PCR products were analyzed on 1.50% agarose gels, purified, and then confirmed as authentic by sequencing. Samples that failed to amplify are defined herein as untyped.

### Phylogenetic analysis

All the hexon gene sequences obtained by nested PCR together with the 26 HAdV strain sequences available in GenBank were used for the phylogenetic analyses. The phylogenetic tree was constructed using molecular evolutionary genetics analysis (Mega) 5.0 and evaluated using 100 bootstrap replicates to verify the HAdV genotypes. Positive PCR products were named according to the corresponding serial numbers of the specimens.

### Statistical analysis

The HAdV detection rates for the different populations and different seasons were compared by a χ2 test, and the relationship between vomiting and HAdV types was statistically analyzed by Fisher’s exact probability, with statistical significance set at *P* < 0.05. Vector NTI 11.0 software was used for sequence alignment and Mega 5.0 software for phylogenetic analysis. Epidemiological data were analyzed using statistical product and service solutions (SPSS) 21.0 software.

## Results

### Characteristics of the inpatient children

In total, 1276 NPA specimens were obtained from 1276 children with RTIs at Beijing Friendship Hospital, among which 674 (52.82%) were male and 602 (47.18%) were female (Table 1), all children survived, and the age range was from 1 day to 14 years of age with a median age of 3 years. From them, 897 (70.30%) patients were younger than 5 years old.

**Table 1:** HAdV infections in children of different ages and gender with RTIs

Variable	Number of children	Number of children positive for HAdV	Percentage children positive for HAdV (%)	*P* value
Age (years)				0.008
≤ 1	314	10	3.18	
> 1 to ≤2	140	9	6.43	
> 2 to ≤3	109	10	9.17	
> 3 to ≤4	193	13	6.73	
> 4 to ≤5	141	9	6.38	
> 5 to ≤6	86	11	12.7	
> 6 to ≤14	293	10	3.41	
Gender				0.090
Male	674	45	6.67	
Female	602	27	4.48	
Total	1276	72	5.64	

### Epidemiology of HAdV

**Fig. 1:**
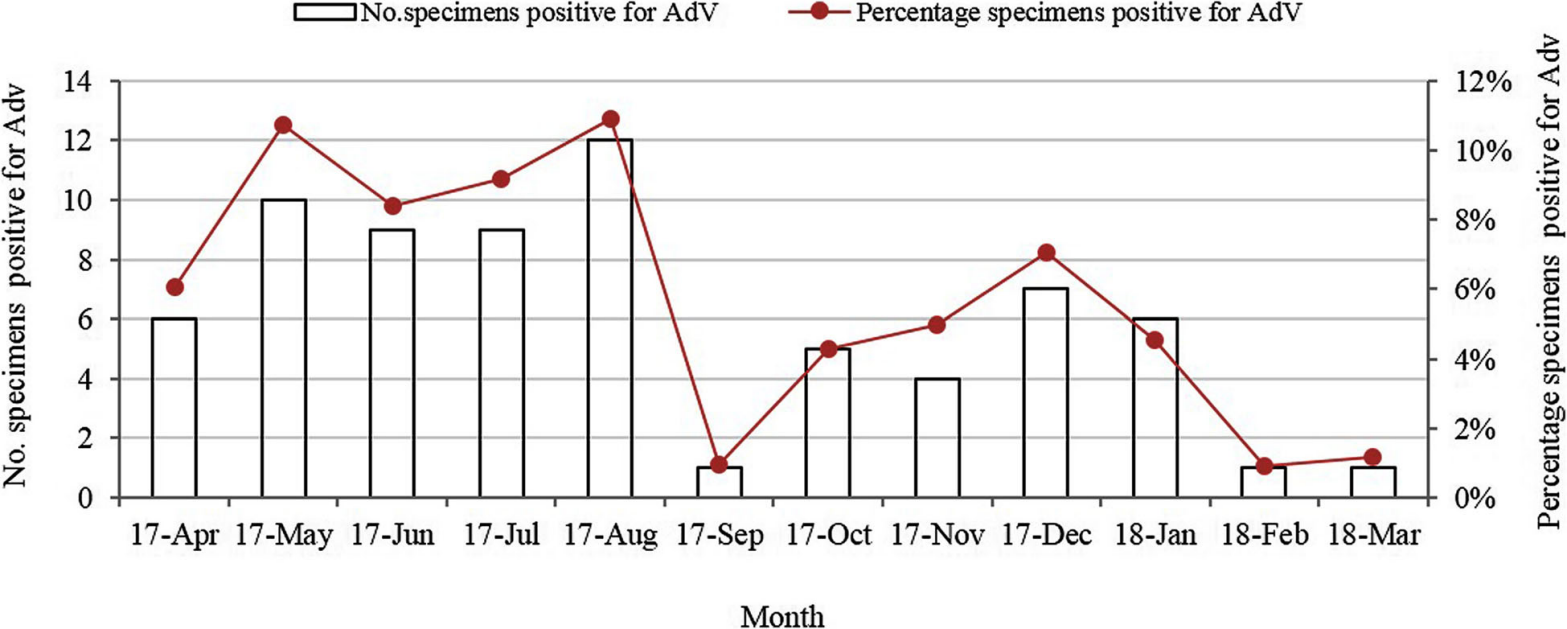
Seasonal distribution of HAdVs in children with RTIs from April 2017 to March 2018

HAdVs were detected in 5.64% (72/1276) of the hospitalized children. As shown in Table 1, among the 72 HAdV-positive patients, 45 (62.50%) were male and 27 (37.50%) were female (a male/female ratio of 1.6:1). No significant difference was observed in males and females in the HAdV-positive cases (*P* = 0.090). HAdV was detected among hospitalized children (< 14 years) with RTIs at Beijing Friendship Hospital between April 2017 and March 2018, and there were significant differences in HAdV detection rates among different age groups (*P* = 0.008). Children under 6 years old accounted for 86.11% (62/72) of the infections. The HAdV detection rate peaked in the > 5 to≤6 years age group (12.79%, 11/86), while those in the ≤1 years old group had a relatively low detection rate of 3.18% (10/314). HAdV was detected in every month throughout the study period from April 2017 to March 2018, peaking in summer. The HAdVs detection frequencies were 5.60% (18/321), 9.52% (30/315), 3.31% (10/302) and 4.14% (14/338) (χ2 = 16.06, *P* = 0.001) in the spring, summer, autumn and winter months, respectively. Additionally, the HAdV detection rate peaked at 10.91% (12/110) in August 2017 (Fig. 1).

### Coinfections with other respiratory viruses

**Fig. 2:**
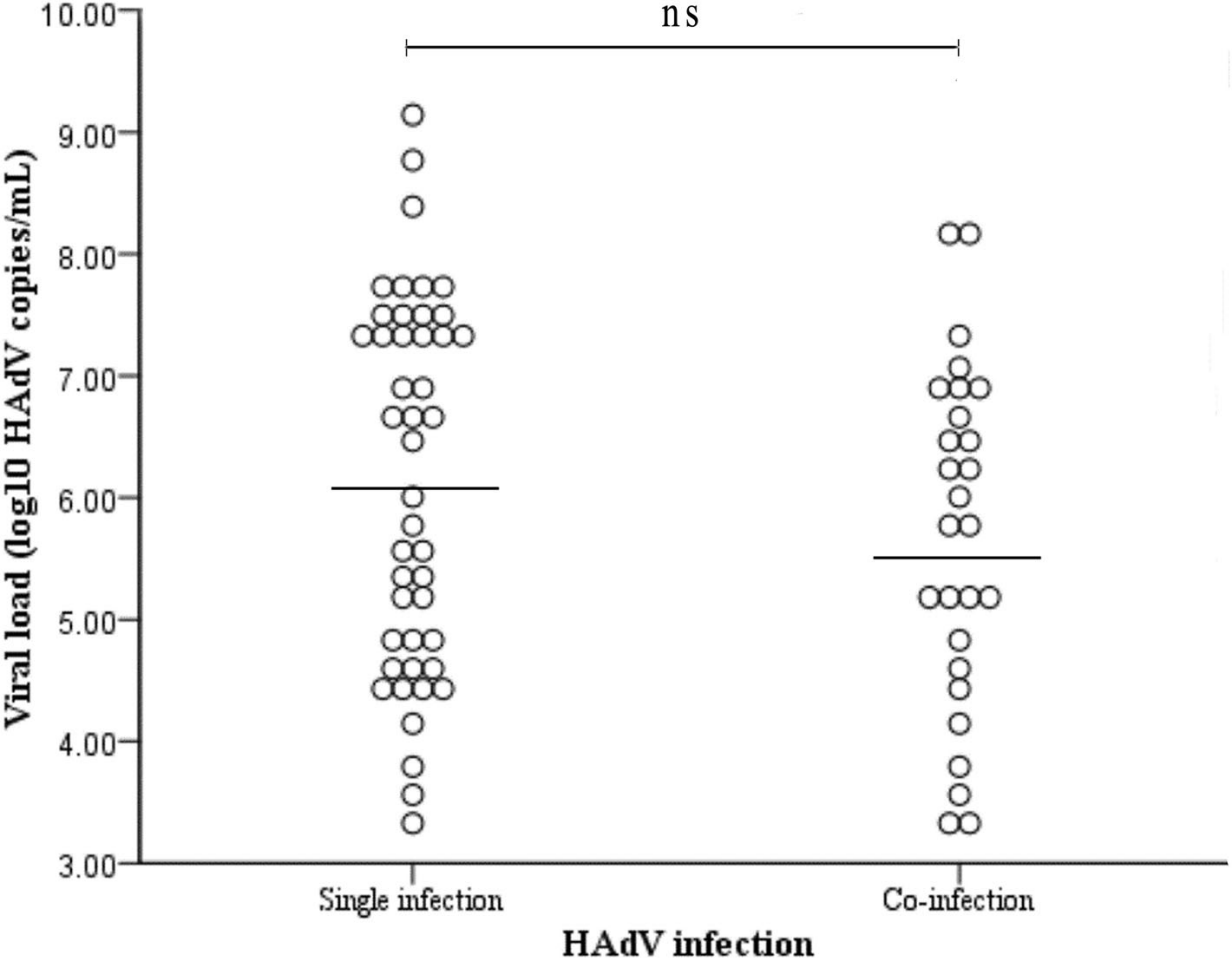
HAdV viral loads in NPA from children with RTIs with or without other respiratory coinfections

Of the 72 HAdV-infected cases, 27 (37.50%) comprised co-infections with other respiratory viruses including 21 (77.78%, 21/27) with one other virus, 3 (11.11%, 3/27) with two other viruses, 2 (7.41%, 2/27) with three other viruses and 1 (3.70%, 1/27) with four other viruses. The most frequently identified mixed infection was PIV (12.50%, 9/72) and HRV (9.70%, 7/72), as shown in Table 2. Among the HAdV-positive specimens, the log number of HAdV genome copies ranged from 3.30 to 9.14 copies per mL of NPA, as determined by the qPCR assay measurements (Fig. 2). The log number for the HAdV genome copies was 6.14 ± 1.52 copies/mL of NPA in the children infected with HAdV only, which is slightly higher than the 5.70 ± 1.39 copies/mL of NPA from those co-infected with HAdV and other respiratory viruses; there was, however, no significant difference in the viral loads between the HAdV mono- and co-infections (*P* = 0.220).

**Table 2:** Co-detection of HAdV and other respiratory viruses

Coinfection	Virus composition	Number of cases
2 Virus (*N* = 21)	HAdV+HRV	7
	HAdV+PIV	5
	HAdV+FluA	3
	HAdV+HCoV	2
	HAdV+RSV	2
	HAdV+HMPV	1
	HAdV+WUPyV	1
3 Viruses (*N* = 3)	HAdV+PIV + RSV	2
	HAdV+HCoV+FluA	1
4 Viruses (N = 2)	HAdV+PIV + HCoV+HMPV	1
	HAdV+RSV + HBoV+WUPyV	1
5 Viruses (*N* = 1)	HAdV+PIV + RSV + HMPV+WUPyV	1

### Clinical characteristics of the HAdV infections

Sixty-two of the patients (86.11%, 62/72) from the 72 HAdV-positive cases were diagnosed with pneumonia, including 46 with bronchopneumonia, 4 with asthmatic bronchitis, 3 with refractory bronchopneumonia, 8 with pneumonia (3 with mycoplasma pneumonia, 3 with bacterial pneumonia and 2 with viral pneumonia) and 1 with refractory pneumonia. Viral pneumonia and bacterial pneumonia accounts for 3.23% (2/62) and 4.84% (3/62) of the pneumonia, respectively. In addition, the remaining 10 cases were diagnosed with acute tonsillitis (5), acute upper respiratory infection (2), acute bronchitis (2), as well as 1 with Epstein-Barr virus-associated infectious mononucleosis. The main clinical signs in the HAdV-positive patients were fever (97.22%, 70/72) and cough (75%, 54/72), while the other clinical presentation were vomiting (18.06%, 13/72), gasping (15.28%, 11/72), diarrhea (4.17%, 3/72) and angina (5.56%, 4/72). Furthemore, vomiting didn’t associate with specific HAdV types by Fisher’s exact probability (*P* = 0.069). In this study, of the 72 HAdV-positive children with RTIs, 4 (5.56%, 4/72) had asthma, the other 68 had no pre-existing chronic conditions.

### HAdV genotyping and phylogenetic analysis

**Fig. 3:**
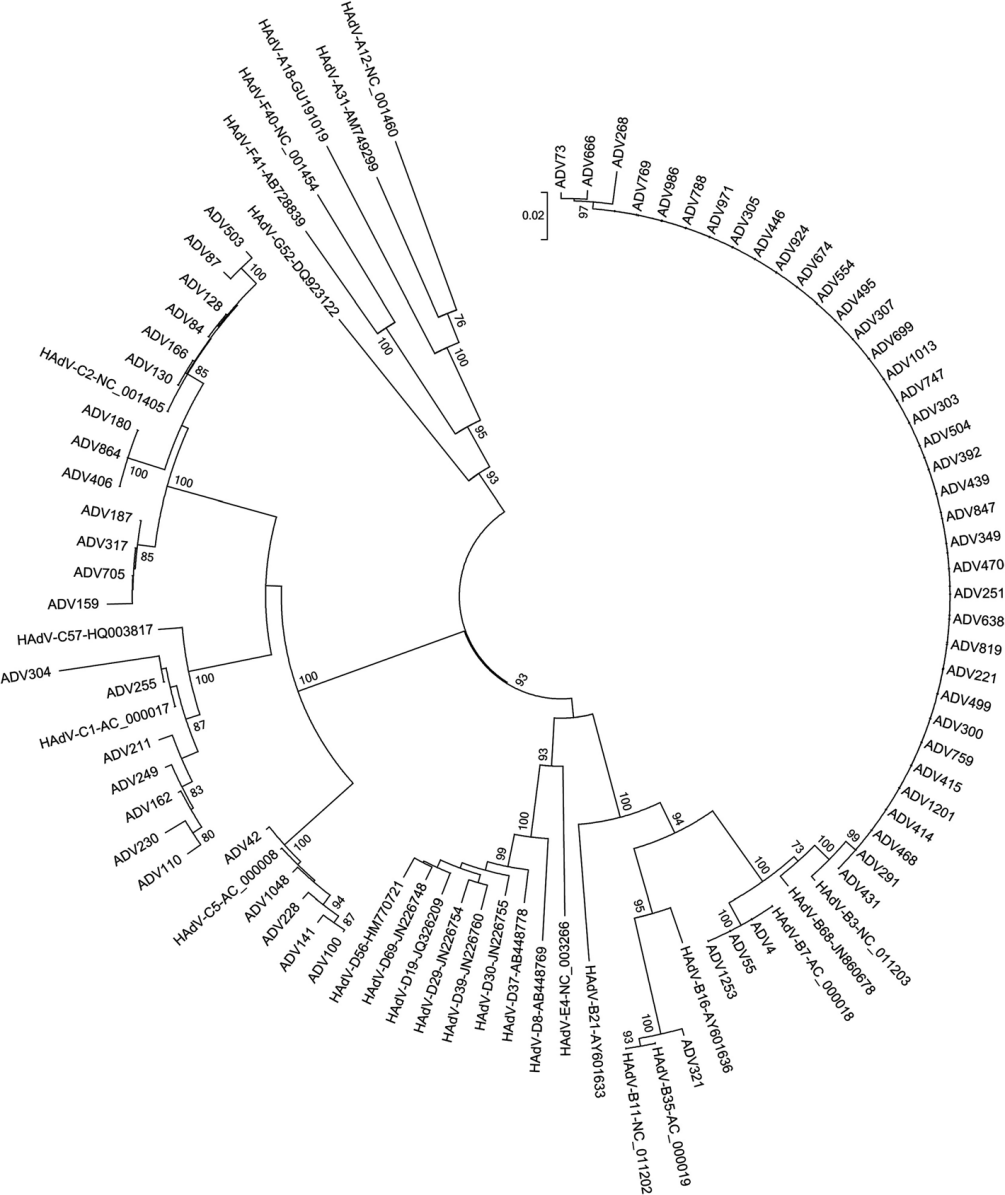
Phylogenetic analysis of nucleic acid sequences of HAdV hexon gene (956-bp) compared with reference strains

Basing on the 956-bp fragment of the hexon gene amplified by nested-PCR, 66 HAdV-positive samples were sequenced and successfully genotyped. The phylogenetic analyses indicated that 41 cases belonged to species B (HAdV-B3, HAdV-B7, and HAdV-B35) and 25 belonged to species C (HAdV-C2, HAdV-C1, and HAdV-C5) (Table 3). Six of the samples failed to be typed. The above-mentioned results indicate that species B and C (at least 6 HAdV genotypes) circulated simultaneously in Beijing, and that HAdV-B3 was the most prevalent genotype, followed by HAdV-C2 (Fig. 3).

**Table 3:** HAdV species and types detected from positive NPA samples

Species (%)	types	Number of specimens (%)
B (62.12, 41/66)	3	37 (56.06, 37/66)
	7	3 (4.55, 3/66)
	35	1 (1.52, 1/66)
C (37.88, 25/66)	2	13 (19.70, 13/66)
	1	7 (10.61, 7/66)
	5	5 (7.58, 5/66)
Untyped	–	6

## Discussion

The present study was carried out between April 2017 and March 2018 among hospitalized children with RTIs in Beijing, China. Herein, we describe (i) the prevalence of HAdVs in hospitalized children with RTIs presenting at Beijing Friendship Hospital during a one-year period; (ii) the clinical spectrum of the HAdV-positive RTI patients; (iii) the viral co-pathogens present in the HAdV infections; and (iv) the genetic diversity of the HAdVs.

The clinical characteristics of the RTIs caused by HAdV are very similar to those of influenza, PIV and other respiratory tract pathogens, making it difficult to clinically diagnose this type of infection. Therefore, effective diagnostic methods are needed for rapid identification and genotyping of HAdV. The qPCR assay used herein was established to detect and quantify HAdV. A total of 1276 NPA specimens were screened for the presence of HAdV and 72 specimens (5.64%, 72/1276) were confirmed to be positive for HAdV, which is consistent with prior reports (1.70–13.90%) [16–18]. The detection rate for HAdV varies from region to region in China, and the rate for hospitalized children with acute lower RTIs in Zhejiang province from 2006 to 2012 was 0.63, and 2.24% in Shenzhen city from 2012 to 2015 [19, 20]. However, it is worth mentioning that such discrepant HAdV detection rates can be caused by methodological differences, the number of patients tested, the periods during which the samples are collected, and even a study’s duration.

Previous studies have shown that HAdV detection rates are positively correlated with the monthly mean temperature and sunshine duration, and negatively correlated with wind speed; in fact, the higher the air temperature, the higher the detection rate [21]. Our study also revealed that HAdV infections occurred throughout the year with the highest prevalence in the summer (9.52%, Jun to Aug), peaking in August (10.91%), which is similar to what has been found in Tianjin, a northern Chinese city, where HAdV infections are concentrated during the summer [22]. However, this finding is discordant with other studies that have reported seasonal peaks for HAdV infections in spring in Northern China and Mexico [23, 24].

We found that the HAdV infections occurred predominantly in children under 6 years of age (86.11%), demonstrating that HAdV is an important pediatric pathogen. Previous studies drew identical conclusions that most children become infected by HAdV at an early age [18, 25, 26]. HAdVs can be easily transmitted through fomites contaminated with infectious body fluids. In our study, the HAdV detection rate (3.18%) was lowest among children under 1 year of age, and the reasons need to be further explored.

Many studies have reported that the most common HAdV species causing RTIs in children are B (B3, B7, B21), C (C1, C2, C5, C6) and E (E4) worldwide [27, 28]. HAdV 2, 3 and 7 are the most prevalent species and are associated with severe pneumonia in China [29, 30]. In the present study, a total of 66 samples were identified and phylogenetically analyzed based on the hexon gene sequence, which revealed six HAdV genotypes and showed that HAdV species B and C were the most prevalent, accounting for 62.12 and 37.88% of isolates, respectively. Similarly to what has been found in Asia by other authors, HAdV-B3 was the most common type (56.06%, 37/66) followed by HAdV-C2 (19.70%, 13/66) and C1 (10.61%, 7/66). HAdV-B3 has been identified as the causative pathogen for severe acute respiratory illness outbreaks in Korea [31], Brazil [32] and Taiwan [33], and it was the main type of respiratory HAdV infections from 1981 to 2002. Moreover, HAdV-B3 was the pathogen causing epidemic respiratory disease outbreaks in Europe, America, and Oceania. Last, HAdV-B3 is known to cause a characteristic syndrome in older children and adults, as manifested by acute pharyngo-conjunctival fever, especially after contact with summer camps and swimming pools [34]. HAdV- C1 and HAdV-C2 have been frequently reported to cause endemic, sporadic and epidemics cases [35, 36].

In the present study, the most common diagnosis (86.11%) in the HAdV-positive cases was pneumonia, with the common signs and symptoms of fever and cough, which is consistent with previous reports [16–18]. HAdV infection is often accompanied by other virus infections. Our study showed that PIV (12.50%, 9/72) was the major co-infecting pathogen identified followed by HRV (9.70%, 7/72). Although the viral load from children mono-infected with HAdV was slightly higher than those co-infected with HAdV and other respiratory viruses, no significant difference was seen between the two groups. The severity of HAdV infection varies according to age, socioeconomic status, environmental status and, above all, the immunological characteristics of the patient. Therefore, the etiological significance of co-infections with HAdV and other respiratory viruses and its association with disease severity require further study. It was reported HAdV can cause more severe illness in immunocompromised patients, so it would be very valuable to know about pre-existing conditions in HAdV-positive children. Except for 4 cases of asthma, there were no other comorbidities in our study. Taken together, our results provide a foundation for further clarification of the role played by HAdVs in RTIs and for defining the clinical and public health significance of HAdV infections.

## Conclusions

This 1-year study documented the prevalence, age distribution, seasonality and molecular epidemiology of HAdV infections among children hospitalized with RTIs at Beiing Friendship Hospital. Our results reveal recent changes in the trends for circulating HAdV genotypes linked to RTIs in Beijing, China, which should be of value for improving the diagnosis of HAdV-related diseases and for developing new detection, treatment and prevention strategies for broad application in clinical laboratories. However, only a 956-bp region of hexon gene was sequenced for genetic analysis in the present study. Therefore, a future full-length genome analysis would help us to understand the extent of genetic variation and any potential recombination that has occurred in the strains referred to above.

## Data Availability

The datasets supporting the conclusions of this article are included within the article.

## Abbreviations

Ct: Cycle threshold
FluA: Influenza A virus
FluB: Influenza B virus
HAdVs: Human adenoviruses
HBoV: Human bocavirus
HCoV: Human coronavirus
HMPV: Human metapneumovirus
HRV: Human rhinovirus
Mega: molecular evolutionary genetics analysis
NPA: Nasopharyngeal aspirate
PIVs: Parainfluenza viruses
qPCR: quantitative real-time polymerase chain reaction
RSV: Respiratory syncytial virus
RTIs: Respiratory tract infections
SPSS: Statistical product and service solutions
WUPyV: WU polyomavirus
